# Targeted and Effective Phage-Based Biocontrol of Black Rot Disease in Broccoli

**DOI:** 10.3390/v18050484

**Published:** 2026-04-22

**Authors:** Miloud Sabri, Khaoula Mektoubi, Orges Cara, Roukia Bougheloum, Angelo De Stradis, Giuseppe Parrella, Toufic Elbeaino

**Affiliations:** 1 International Centre for Advanced Mediterranean Agronomic Studies (CIHEAM of Bari), Via Ceglie 9, Valenzano, 70010 Bari, Italy; miloud.sabri@uit.ac.ma (M.S.); mektoubikhaoula2099@gmail.com (K.M.); org.cara@gmail.com (O.C.); bougheloumroukia@gmail.com (R.B.); 2 National Research Council of Italy (CNR), Institute for Sustainable Plant Protection (IPSP), Via G. Amendola, 122/D, 70126 Bari, Italy; angelo.destradis@cnr.it; 3 National Research Council of Italy (CNR), Institute for Sustainable Plant Protection (IPSP), Piazzale Via Enrico Fermi, 1, Portici, 80055 Naples, Italy; giuseppe.parrella@cnr.it

**Keywords:** *Xanthomonas campestris* pv. *campestris*, sustainable management, phages, *in planta*, brassica-associated bacteria

## Abstract

*Xanthomonas* species are Gram-negative bacterial pathogens responsible for diseases in over 400 plant hosts, including numerous economically important crops such as *Brassica* species. The limited efficacy and environmental concerns associated with chemical control strategies underscore the need for sustainable and targeted alternatives. In this study, we evaluated the suitability and biocontrol efficacy of phages Phi1 and Phi3 to combat *Xanthomonas campestris* pv. *campestris* (*Xcc*) in broccoli plants. Kill-curve assays demonstrated that both phages effectively suppressed *Xcc* growth across a range of multiplicities of infection. Transmission electron microscopy further confirmed their lytic activity, revealing pronounced structural damage to *Xcc* cells following phage treatment, accompanied by the subsequent release of phage progeny. To assess host specificity and biosafety, the phages were tested against 41 bacterial isolates that were isolated and taxonomically characterized from broccoli and cauliflower in this study. Neither Phi1 nor Phi3 exhibited lytic activity against any non-target isolate, indicating high host specificity and minimal risk to the native Brassica-associated microbiota. *In planta* assays demonstrated that the combined application of Phi1 and Phi3 reduced *Xcc*-induced symptom severity in broccoli plants by 80%. Collectively, these results demonstrate that phages Phi1 and Phi3 represent effective and biologically precise agents for the control of black rot disease in Brassica crops.

## 1. Introduction

Black rot, caused by the Gram-negative bacterium *Xanthomonas campestris* pv. *campestris* (*Xcc*), is the main yield-limiting and destructive disease of cruciferous crops worldwide, including cabbage, broccoli, and cauliflower [1,2]. The pathogen is seed-borne and highly transmissible, leading to systemic infection characterized by V-shaped chlorotic lesions, vascular blackening, and severe yield and quality losses [3]. Under warm and humid conditions, black rot epidemics can result in substantial economic damage, particularly in regions with intensive brassica production [4]. In cauliflower, yield reductions of up to 50–60% have been documented, and infected plants show increased susceptibility to secondary pathogens such as *Alternaria* spp., resulting in reduced curd quality and marketability [4,5]. Management of black rot remains challenging. Traditional control strategies, such as the use of copper-based bactericides, crop rotation, and resistant varieties, have had limited success due to the emergence of copper-resistant strains and the lack of broad-spectrum resistance in commercial cultivars [6]. In addition, increasing regulatory pressure on chemical bactericides has highlighted the need for alternative, sustainable disease control approaches.

Bacteriophage (phage)-based control has attracted considerable attention as a promising biocontrol strategy for managing plant pathogenic bacteria. Phages are naturally occurring viruses that specifically infect and lyse bacterial cells, offering a highly targeted, eco-friendly, and residue-free approach to disease management [7]. Phage-based biocontrol strategies have demonstrated efficacy against a range of plant pathogenic bacteria, including *Erwinia*, *Ralstonia*, *Xylella*, *Dickeya*, and *Xanthomonas* species, with consistent and promising outcomes reported under both greenhouse and field conditions [8,9,10,11,12]. In the case of *Xcc*, several studies have highlighted the potential of phages to effectively reduce bacterial populations and disease severity. Notably, a phage cocktail comprising FoX2 and FoX6 significantly reduced *Xcc* populations and disease symptoms under both greenhouse and field conditions, underscoring its potential as a sustainable management strategy [13]. Similarly, foliar application of phage MATE 2 in combination with *Lactococcus lactis* subsp. *lactis* on broccoli achieved a 71% reduction in black rot symptoms, indicating significant synergistic efficacy [14]. The observed synergism among phages and with other antibacterial agents substantiates their utility as highly specific and effective biocontrol agents for the management of plant bacterial pathogens. While previous studies have demonstrated the efficacy of phage-based approaches against *Xcc*, the present study extends this work by systematically assessing the specificity, host range, and biosafety profile of a phage cocktail within an ecologically relevant Brassica-associated context. To achieve this, we first isolated and characterized a collection of bacterial strains associated with Brassica crops (cauliflower and broccoli), representing the natural microbial communities inhabiting the host plant environment. These isolates were then used to comprehensively evaluate the host range of the phage cocktail beyond *Xcc*, with particular attention to potential off-target interactions with non-target bacterial members of the Brassica microbiota. Based on this framework, the study evaluates the biocontrol potential of the phage cocktail against *Xcc* and its suitability as a targeted and sustainable strategy for managing black rot disease in broccoli.

## 2. Materials and Methods

### 2.1. Bacteriophage, Bacterial Strains, and Culture Conditions

*Xanthomonas campestris* pv. *campestris* strain CFBP 1710 was cultured at 28 °C in liquid yeast extract–peptone–glucose (YPG) broth (5.0 g/L yeast extract, 5.0 g/L peptone, 10.0 g/L glucose) or on yeast extract–peptone–glucose agar (YPGA; YPG supplemented with 1.5% agar). Two previously characterized bacteriophages, Xylella phage Phi1 (GenBank accession number PX095379) and Xylella phage Phi3 (GenBank accession number PX095380), both reported to infect *Xylella fastidiosa* and *Xcc* [15], were employed in this study. Phi1 is a podovirus with a genome size of 44,345 bp, whereas Phi3 is a siphovirus with a genome size of 55,413 bp. Both phages undergo a strictly lytic infection cycle, with no detectable temperate markers, virulence factors, or antimicrobial resistance genes, and their suitability as biocontrol agents has been previously evaluated [15].

### 2.2. Assessment of Phi1 and Phi3 Lytic Activity Against Xcc

The lytic activity of Phi1 and Phi3 against *Xcc* was evaluated using transmission electron microscopy (TEM) and spot assays. Briefly, *Xcc* cultures were infected with Phi1 and Phi3 at a multiplicity of infection (MOI) of 1 and incubated at 28 °C for 24 h. Following infection, cells were adsorbed for 2 min onto carbon-coated copper/rhodium grids, rinsed with distilled water, and negatively stained with 0.5% (*w*/*v*) aqueous UA-Zero EM stain (Agar Scientific, Essex, UK). Grids were examined using an FEI Morgagni 282D transmission electron microscope operated at 80 kV (FEI MORGAGNI 282D, Hillsboro, OR, USA).

For spot assays, 200 μL of an *Xcc* suspension (10^8^ CFU/mL) was mixed with 6 mL of YPG soft agar (YPG supplemented with 0.7% agar), poured onto YPGA plates, and allowed to solidify under a laminar flow hood. Subsequently, 10 μL drops of phage suspensions at different concentrations (10^8^, 10^7^, 10^6^, 10^5^, and 10^4^ PFU/mL) were spotted onto the agar surface. Plates were incubated at 28 °C for 24 h, and bacteriolytic activity was perceived as a clear inhibition zone of *Xcc* growth.

### 2.3. Assessment of the Optimal Multiplicity of Infection

The optimal multiplicity of infection for Phi1 and Phi3 was determined by infecting *Xcc* suspensions across a range of MOIs and monitoring bacterial growth over time. Briefly, phage suspensions (200 μL) were mixed with *Xcc* suspensions (200 μL) to obtain final MOIs of 1, 0.1, 0.01, and 0.001. Each mixture was inoculated into 2 mL of YPG broth in triplicate and incubated at 28 °C for 24 h. Bacterial growth was monitored by measuring optical density at 600 nm (OD_600_) at 0, 2, 4, 8, 20, and 24 h using a UV–Vis spectrophotometer (ThermoFisher Scientific, Waltham, MA, USA).

### 2.4. Broccoli- and Cauliflower-Associated Bacteria and Off-Target Testing

To evaluate the potential effects of Phi1 and Phi3 on non-target plant-associated bacteria, bacterial isolates were obtained from cauliflower (*Brassica oleracea* var. *botrytis*) and broccoli (*Brassica oleracea* var. *italica*) plants. Three whole plants of each species were collected from an orchard in Valenzano (Bari, Italy). Stalk and leaf tissues were ground in 5 mL of sterile distilled water and serially diluted. Aliquots of 50 µL from each dilution were plated onto YPGA plates and incubated at 28 °C for 48 h. Morphologically distinct colonies were selected and preserved in 25% (*v*/*v*) glycerol in YPG broth at −80 °C. Genomic DNA from all isolates was extracted using the CTAB method [16]. The 16S rRNA gene was amplified by PCR using universal primers 27F (5′-AGAGTTTGATCCTGGCTCAG-3′) and 1492R (5′-GGTTACCTTGTTACGACTT-3′) [17], followed by Sanger sequencing for taxonomic identification.

The sensitivity of bacterial isolates to bacteriophages was evaluated using the spot assay method, as described above. Four titers (10^8^, 10^7^, 10^6^, and 10^5^ PFU/mL) of each phage were tested, and bacterial isolates showing no visible lysis zones at any titer were considered phage-insensitive.

### 2.5. Evaluation of the Biocontrol Efficacy of Phages Phi1 and Phi3 Against Black Rot in Broccoli Plants

The preventive biocontrol efficacy of phages Phi1 and Phi3, applied individually and in combination, against black rot disease was evaluated using an *in planta* assay on one-month-old broccoli plants, following the methodology described by Sabri et al. (2024) [14]. Briefly, four leaves per plant were gently wounded using a multi-needle pricker to facilitate pathogen entry, after which plants were sprayed with a phage suspension (3 mL per plant; 10^8^ PFU/mL). After 24 h, plants were inoculated with *Xcc* by foliar spraying (0.66 mL per plant; OD_600_ = 0.2). Plants inoculated with *Xcc* alone and plants treated with sterile water served as positive and negative controls, respectively. Each treatment consisted of 10 plants, with four leaves inoculated per plant, resulting in a total of 40 biological replicates per treatment. Following inoculation, plants were maintained in a greenhouse under controlled environmental conditions (25 ± 2 °C and approximately 70% relative humidity). Disease severity was assessed 14 days post-infection (dpi) by visually estimating the necrotic area on inoculated leaves using a six-level ordinal disease scale: 0 = no visible symptoms; 1 = early symptoms, including small yellow lesions or initial chlorosis; 2 = approximately one-quarter of the leaf area exhibiting chlorosis and lesion development; 3 = approximately one-third of the leaf area affected, with pronounced yellowing and necrotic lesions; 4 = one-half or more of the leaf area showing extensive chlorosis and necrosis; and 5 = the entire leaf displaying severe symptoms, including complete chlorosis, necrosis, and tissue collapse.

The efficacy of phage treatments in reducing black rot severity was calculated using the following formula:

$$Efficacy(\%)=100-(\frac{\sum \left(P\times T\right)}{\sum \left(P\times C\right)}\;\times 100)$$

where P = severity score, T = number of inoculated leaves of the treated plants having the same severity score, and C = number of inoculated leaves of the positive control plants having the same severity score.

To quantify the viable *Xcc* load in treated and untreated leaves, a viability-quantitative PCR (V-qPCR) assay using PMAxx^TM^ (Biotium, Rome, Italy) was performed. For each plant (10 plants per treatment), 1 g of inoculated tissue was collected and homogenized in sterile water. Samples were treated with PMAxx^TM^ at a final concentration of 7.5 μM, incubated in the dark at room temperature for 8 min, and photoactivated for 15 min using the PMA-Lite^TM^ LED Photolysis Device (Biotium, Rome, Italy). Genomic DNA of all samples was extracted using the CTAB method [16]. V-qPCR was performed using a CFX96 Real-Time PCR System (Bio-Rad, Milan, Italy) with *Xcc*-specific primers HrcCF2 (5′-CGTGTGGATGTGCAGACC-3′) and HrcCR2 (5′-CAGATCTGTCTGATCGGTGTCG-3′) under the conditions previously reported in Papaianni et al. (2020) [18]. The relative quantity of bacterial DNA in each sample was determined using the relative quantification method with an external calibrator [19], calculated as follows:

$$\mathrm{Relative}\;\mathrm{Quantity}=2^{-\Delta Cq},\Delta Cq=Cq_{\mathrm{Sample}}-Cq_{\mathrm{Reference}\;\mathrm{Standard}}$$

### 2.6. Persistence of Phages Phi1 and Phi3 in Broccoli Plants

The persistence of phages Phi1 and Phi3 in broccoli plants at 14 dpi was evaluated. For each phage, three leaves were collected, homogenized in 4 mL of sterile water, and centrifuged at 1000× *g* for 5 min. The supernatants were filtered through 0.22 μm syringe filters (Acrodisc^®^, Merck, Italy) and enriched by mixing 500 μL of filtrate with 200 μL of an *Xcc* suspension (OD_600_ = 0.2) in 2 mL of YPG broth, followed by incubation at 28 °C for 24 h. After enrichment, samples were re-filtered and analyzed by spot assay on *Xcc*, as previously described, and by using Phi1-specific primers (Phi1-F: 5′-AGCGATCCGAACTTGCAGAA-3′; Phi1-R: 5′-AAGGAGAACACCTTCGCGTT-3′) and Phi3-specific primers (Phi3-F: 5′-TTTATTCATGGTCGCCGCCT-3′; Phi3-R: 5′-CAGGGCCGGAAGTACCTTTT-3′), designed in this study using Geneious Prime 2025.1.1 (Biomatters Ltd., San Diego, CA, USA). Phi1-specific primers target a DNA polymerase gene region (10,285–10,715), while Phi3-specific primers target a gene encoding a hypothetical protein (44,105–44,467), both selected as unique genomic regions to ensure phage-specific detection and amplification. PCR amplification was performed with an initial denaturation at 94 °C for 5 min, followed by 35 cycles of 94 °C for 30 s, 60 °C for 30 s, and 72 °C for 30 s, and a final extension at 72 °C for 5 min.

### 2.7. Statistical Data Analysis

Statistical analyses were performed using RStudio vers. 2026.01. Data normality was assessed using the Shapiro–Wilk test, which revealed a significant deviation from normality. Consequently, the nonparametric Kruskal–Wallis test was applied to compare treatments. Post hoc pairwise comparisons were carried out using Dunn’s test with Bonferroni correction to adjust for multiple comparisons. Statistically significant groupings were determined using the multcompLetters function. Mean values and standard deviations were calculated for each treatment for graphical representation.

## 3. Results

### 3.1. Lytic Activity of Phages Phi1 and Phi3 Against Xcc

**Figure 1:**
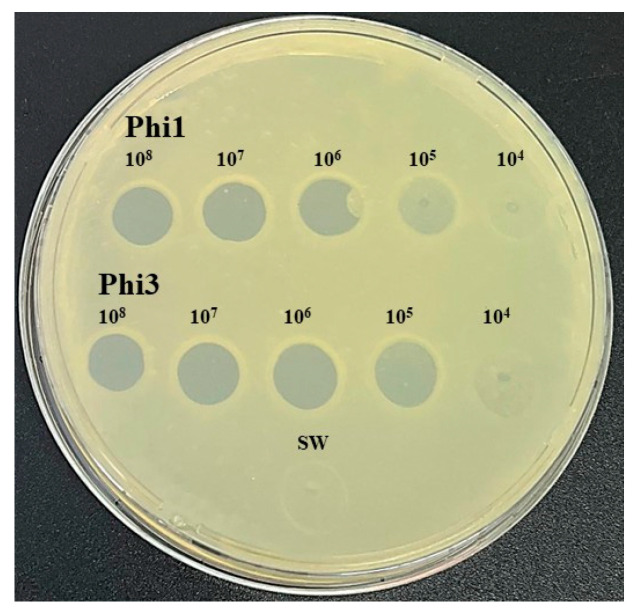
Spot assay demonstrating the bacteriolytic activity of phages Phi1 and Phi3 against *Xanthomonas campestris* pv. *campestris*. Phage suspensions were tested at titers ranging from 10^8^ to 10^4^ PFU/mL. Sterile water was used as the negative control.

**Figure 2:**
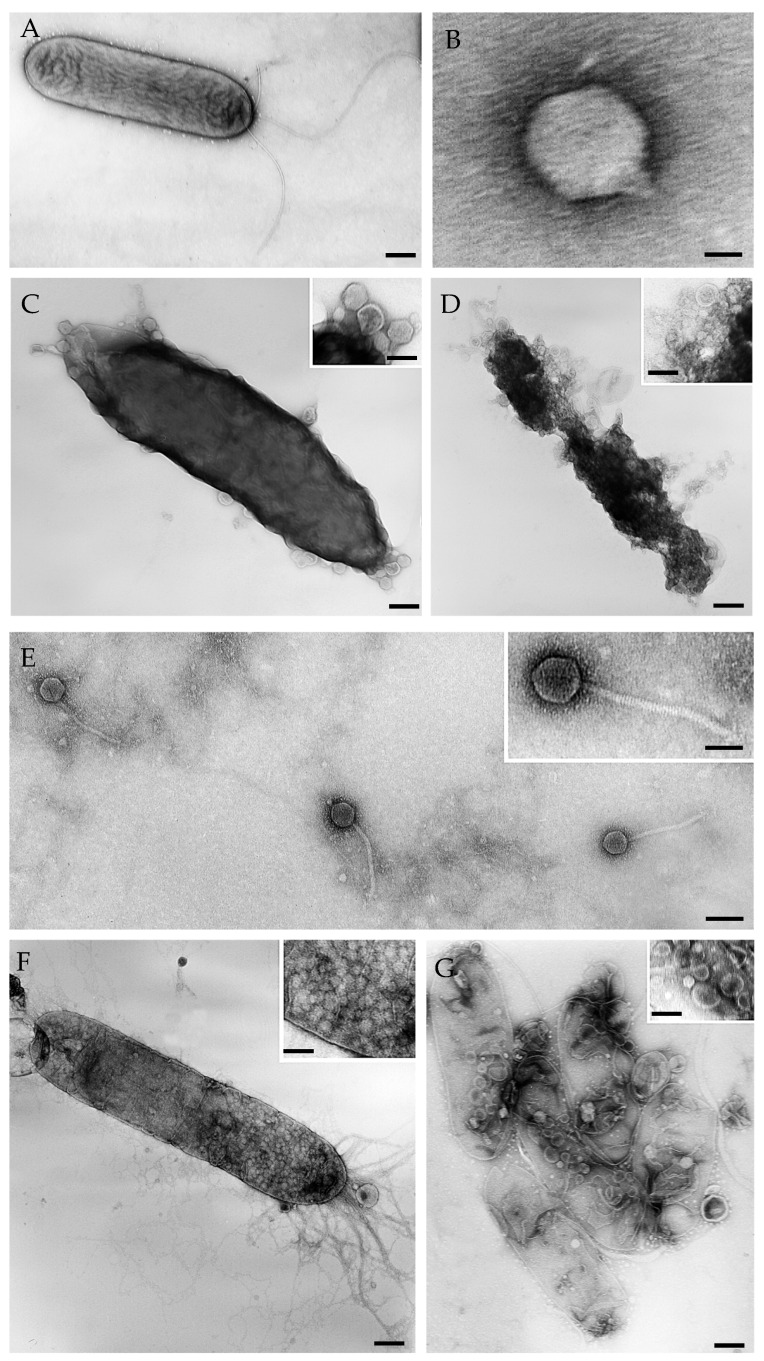
Transmission electron micrographs of phages Phi1 and Phi3 and their effects on *Xanthomonas campestris* pv. *campestris* cells: (**A**) Untreated *Xcc* cells used as a control (scale bar = 100 nm). (**B**) Phi1 virion exhibiting a podovirus morphotype (scale bar = 25 nm). (**C**,**D**) *Xcc* cells treated with Phi1, showing cell lysis with release of progeny virions (scale bars = 50 nm, inset = 25 nm). (**E**) Phi3 virions exhibiting a siphovirus morphotype (scale bar = 100 nm; inset = 50 nm). (**F**,**G**) *Xcc* cells treated with Phi3, showing phage replication and bacterial cell lysis (scale bar = 100 nm; inset F = 50 nm, G = 25 nm).

Pronounced bacteriolytic activity against *Xcc* was observed for both Phi1 and Phi3. In spot assays, distinct zones of lysis were observed at phage titers ranging from 10^8^ to 10^5^ PFU/mL, whereas lytic activity was markedly diminished at 10^4^ PFU/mL, where only weak and partial lysis zones were evident (Figure 1). Transmission electron micrographs clearly illustrate the lytic potential of Phi1 and Phi3 against *Xcc*, revealing intracellular phage replication and subsequent bacterial cell disruption accompanied by the release of progeny virions (Figure 2). Together, these results demonstrate that both phages efficiently infect and lyse *Xcc*, highlighting their potential as promising biocontrol candidates.

### 3.2. Optimal Multiplicity of Infection

**Figure 3:**
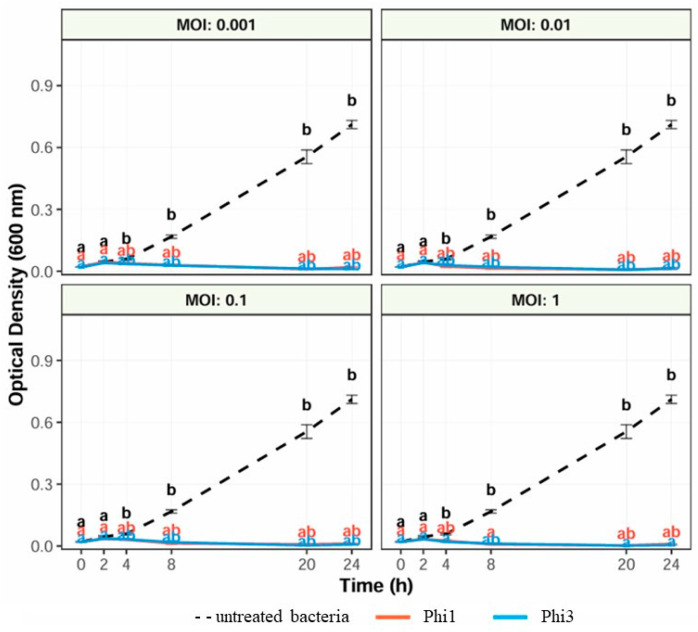
Bacterial killing curves illustrating the inhibitory effect of phages Phi1 and Phi3 at different multiplicities of infection (MOIs) against *Xanthomonas campestris* pv. *campestris*. Each data point represents the mean of three biological replicates, and error bars indicate the standard deviation (SD). Different lowercase letters (a, b, ab) above the data points denote statistically significant differences among treatments at the corresponding time point, as determined by Dunn’s post hoc test (*p* < 0.05).

The ability of Phi1 and Phi3 to control *Xcc* growth in YPG broth was assessed at different MOIs. Untreated *Xcc* cultures exhibited a typical growth pattern, reaching an OD_600_ value exceeding 0.7 after 24 h (Figure 3). In contrast, treatment with either phage resulted in a pronounced inhibition of bacterial growth across all tested MOIs, maintaining optical density values at minimal levels throughout the incubation period (Figure 3). No statistically significant differences were observed among the tested MOIs, indicating that both phages exert strong antibacterial activity over a broad range of infection ratios. These findings are consistent with previous observations and further support the potential of Phi1 and Phi3 as effective biocontrol agents for the management of black rot disease.

### 3.3. Off-Target Effects of Phi1 and Phi3

A total of 41 bacterial strains were isolated from broccoli and cauliflower (Table 1), comprising 14 isolates from broccoli and 27 from cauliflower. These isolates represented a diverse range of species, highlighting the substantial bacterial diversity associated with these crops. The majority belonged to well-characterized plant-associated beneficial species, including *Pseudomonas putida*, *Pseudomonas fluorescens*, *Pseudomonas argentinensis*, *Bacillus siamensis*, *Pantoea agglomerans*, and *Pseudomonas fulva*. A subset of isolates corresponded to marine-associated species, such as *Shewanella baltica* and *Pseudomonas plecoglossicida*, the latter being a known fish pathogen.

All isolates were tested to assess the potential off-target effects of the Phi1 and Phi3 phages, given that broccoli and cauliflower are hosts of *Xcc*. Neither Phi1 nor Phi3 exhibited lytic activity against any of the isolated strains, supporting their safety and specificity as biocontrol agents against *Xcc* in these crops. Additionally, the taxonomic diversity of the isolates provides a valuable reference for the bacteriome of broccoli and cauliflower, forming a foundation for future studies on plant-associated microbial communities and their functional roles.

### 3.4. In Planta Biocontrol Efficacy of Phi1 and Phi3 Against Black Rot Disease

**Figure 4:**
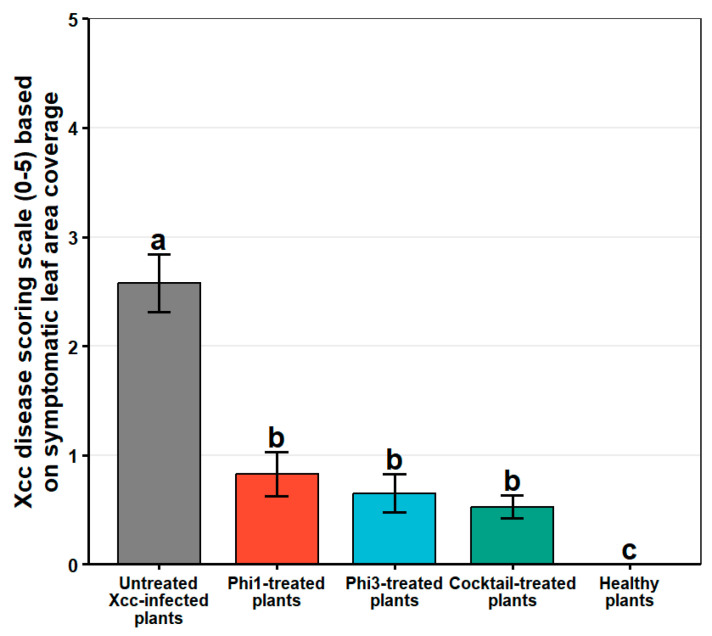
Disease severity of *Xanthomonas campestris* pv. *campestris* on broccoli leaves treated with Phi1 and Phi3, applied individually or as a cocktail. Error bars represent the standard error of the mean (SEM) (*n* = 40). Different letters above the bars indicate statistically significant differences among treatments, as determined by the Kruskal–Wallis test followed by Dunn’s post hoc multiple-comparison test (*p* < 0.001).

**Figure 5:**
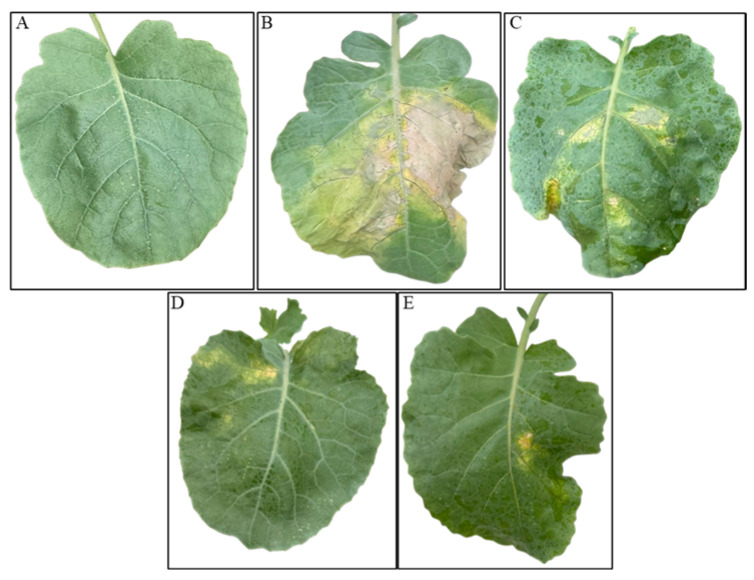
*In planta* assays showing the biocontrol efficacy of Phi1 and Phi3 phages against *Xcc* infection in broccoli plants at 14 dpi: (**A**) healthy broccoli leaf showing no symptoms; (**B**) untreated *Xcc*-infected leaf exhibiting extensive chlorosis and necrosis; (**C**) Phi1-treated *Xcc*-infected leaf showing approximately one-third of the leaf area affected by chlorosis and lesion development; (**D**) Phi3-treated *Xcc*-infected leaf showing approximately one-quarter of the leaf area affected by chlorosis and lesion development; (**E**) phage cocktail-treated *Xcc*-infected leaf exhibiting initial chlorosis. A single representative leaf was photographed for each treatment group based on the corresponding disease severity score.

**Figure 6:**
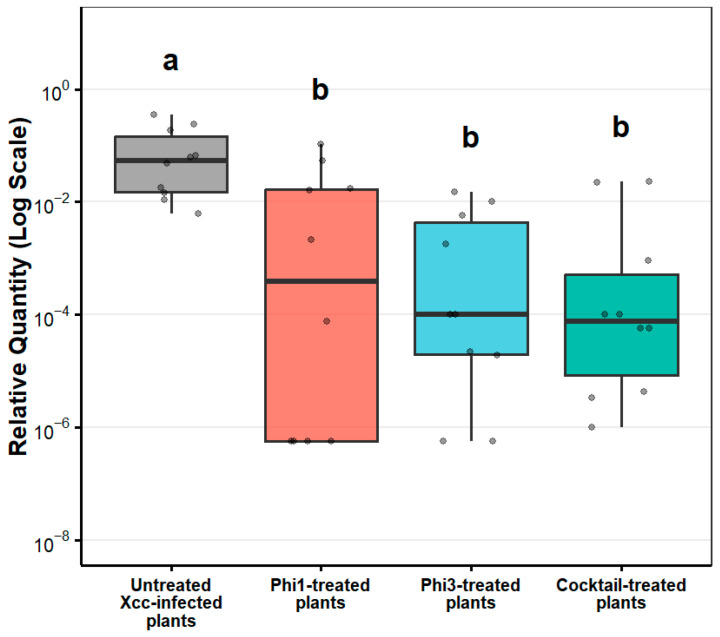
Relative quantity of *Xanthomonas campestris* pv. *campestris* DNA in broccoli leaves following phage treatments. Letters above the boxes indicate statistical groupings determined by a Kruskal–Wallis test followed by Dunn’s post hoc test (*p* < 0.005).

The efficacy of Phi1 and Phi3, applied individually or in combination, in controlling black rot symptoms in broccoli was evaluated at 14 dpi based on symptom severity scores. Statistical analysis using the Kruskal–Wallis test revealed a highly significant difference among treatments (χ^2^(4) = 77.7, *p* < 0.001) (Figure 4). The phage cocktail achieved the greatest reduction in disease severity, resulting in an 80% decrease in symptom development, whereas individual applications of Phi1 and Phi3 led to reductions of 68% and 75%, respectively (Figure 4 and Figure 5). Despite these numerical differences, post hoc comparisons indicated no statistically significant differences among the phage-treated groups, demonstrating comparable efficacy of individual and combined treatments in controlling disease progression. These findings were further supported by v-qPCR analysis. Relative quantification of bacterial DNA revealed significant differences in bacterial load between treated and untreated infected leaves (*p* < 0.005) (Figure 6). The untreated control exhibited a mean quantification cycle (Cq) value of 23.65, whereas phage-treated leaves showed significantly delayed amplification, with mean Cq values ranging from 31.73 to 32.35. This substantial shift in Cq values indicates a marked reduction in viable bacterial populations following phage application. Collectively, the phenotypic and molecular data provide robust evidence that both Phi1 and Phi3, whether applied individually or as a cocktail, exert a strong biocontrol effect against *Xcc* in broccoli plants.

**Figure 7:**
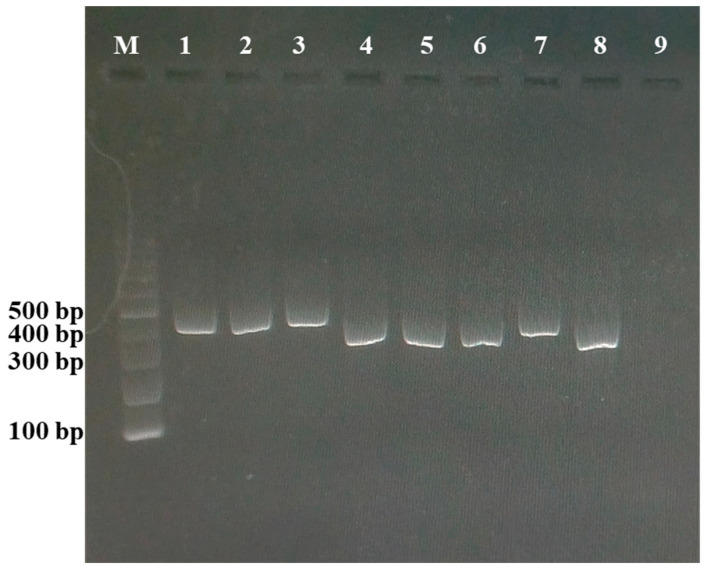
Agarose gel electrophoresis showing PCR products amplified from genomic DNA of Phi1 and Phi3 phages reisolated from broccoli plants 14 dpi. Lane M: 100 bp DNA ladder. Lanes 1–3: Phi1 reisolated from Phi1-treated plants. Lanes 4–6: Phi3 reisolated from Phi3-treated plants. Lanes 7 and 8: Phi1 and Phi3 used as positive controls, respectively. Lane 9: sterile water used as a negative control reaction.

At the end of the experiment, Phi1 and Phi3 were successfully reisolated from treated broccoli plants. The recovered phages retained their lytic activity against *Xcc*, as confirmed by spot assay, and their identity was verified by PCR analysis (Figure 7). These findings demonstrate that both phages persisted within plant tissues throughout the experimental period while maintaining infectivity. Collectively, these results further substantiate the suitability of Phi1 and Phi3 as effective biocontrol agents for the management of black rot disease.

## 4. Discussion

Phages are increasingly recognized as promising biological agents for the management of bacterial diseases in agriculture. Over the past decade, substantial research efforts have been dedicated to characterizing phage diversity, understanding phage-host dynamics, optimizing formulation strategies, and assessing performance under greenhouse and field conditions. This growing body of work reflects a global shift toward environmentally sustainable plant protection strategies, particularly in response to the declining efficacy of conventional bactericides and growing concerns regarding antimicrobial resistance and environmental impact. The present study contributes to this expanding field by providing further evidence that lytic phages can serve as precise, eco-friendly, and effective agents for managing economically significant bacterial plant diseases. In this context, the strictly lytic phages Phi1 and Phi3, whose whole-genome analyses previously confirmed the absence of lysogeny-associated genes, virulence factors, and antimicrobial resistance determinants [15], were evaluated for their suitability and biocontrol potential in broccoli, a major Brassica crop of global economic significance.

In vitro assays showed that both phages significantly suppressed bacterial growth across multiple MOIs, indicating robust infectivity and replication dynamics. The ability to suppress bacterial populations across a range of MOIs is particularly important for field applications, where phage-to-host ratios fluctuate due to environmental factors. Transmission electron microscopy confirmed the lytic activity of Phi1 and Phi3 against *Xcc*, revealing pronounced structural disruption of infected cells followed by the release of progeny virions. These observations are consistent with a strictly lytic replication cycle, a fundamental requirement for their safe use in biocontrol applications. Ecological safety was further assessed, showing that neither phage exhibited lytic activity against any of the 41 bacterial isolates obtained from broccoli and cauliflower, the primary hosts of *Xcc*. This high specificity minimizes the disruption of beneficial Brassica-associated microbiota, which contributes to plant health, nutrient cycling, and natural disease suppression, thus providing a clear advantage over broad-spectrum chemical treatments. Moreover, the characterization of 41 bacterial isolates from broccoli and cauliflower provides key insights into the native microbiota of these crops. Taxonomic analysis revealed a diverse community, including well-known plant-beneficial species such as *Pseudomonas putida*, *Pseudomonas fluorescens*, *Bacillus siamensis*, and *Pantoea agglomerans*, as well as less common or environmental taxa. Defining this microbial baseline is important for future investigations aimed at leveraging microbial interactions to enhance plant health, promote sustainable crop production, and reduce dependency on chemical inputs.

Importantly, *in planta* assays demonstrated that both Phi1 and Phi3 effectively suppressed black rot disease, with a trend toward enhanced efficacy when applied in combination, although the increase did not reach statistical significance. The apparent advantage of the phage cocktail likely arises from complementary lytic activity and a reduced likelihood of resistance development within *Xcc* populations. These observations are consistent with previous studies, such as the study by Holtappels et al. [13], which reported that a cocktail of FoX2 and FoX6 effectively controlled *Xcc* infection in cauliflower. In line with our findings, studies on bacteriophages infecting other *Xanthomonas* species have demonstrated that phage cocktails can enhance biocontrol efficacy and broaden host range. For instance, two lytic phages (pXoo2106 and pXoo2107) targeting *Xanthomonas oryzae* pv. *oryzae* and pv. *oryzicola* were shown to infect multiple strains across both pathovars and, when applied as a cocktail, significantly reduced bacterial populations and disease symptoms in rice [20]. Similarly, lytic phages Φ16, Φ17A, and Φ31 targeting *Xanthomonas axonopodis* pv. *allii* were isolated and evaluated for biocontrol of bacterial leaf blight in Welsh onion, where the phage cocktail effectively reduced disease severity under greenhouse and field conditions, with performance comparable to chemical control and associated with significant yield improvement [21]. Furthermore, the demonstrated persistence and sustained infectivity of Phi1 and Phi3 in broccoli plants, coupled with their ease of application via foliar spraying, further support their suitability as practical and effective biocontrol agents against *Xcc*.

## 5. Conclusions

Brassica vegetables, including broccoli and cauliflower, pose unique challenges for disease management due to their edible, exposed tissues and short growth cycles, which constrain repeated chemical applications. Conventional bactericides raise significant food safety and environmental concerns in these crops. In contrast, phage-based interventions offer a natural, host-specific alternative that poses no known risk to eukaryotic cells, making them particularly suitable for protecting edible plant tissues. The combination of targeted efficacy, ecological safety, and practical applicability underscores the promise of Phi1 and Phi3 as sustainable and eco-friendly tools. Nevertheless, further studies are warranted to optimize phage formulations, monitor potential resistance development, evaluate persistence under field conditions, and integrate phage applications within existing integrated pest management frameworks.

## Figures and Tables

**Table 1:** Bacterial isolates recovered from broccoli and cauliflower for off-target effects assessment of Phi1 and Phi3.

Isolate ID	Isolation Source	Closest Species Match (BLASTX, v2.17.0)	Similarity (%)	Coverage (%)	GenBank Accession (Closest Match)	GenBank Accession (This Study)
BRCL2	Broccoli	*Pantoea dispersa*	99.00	100	GQ246183.1	PZ161046
BRCL3	Broccoli	*Pseudomonas* sp.	99.13	100	MH780493.1	PZ161047
BRCL4	Broccoli	*Pantoea* sp.	99.08	100	AP035945.1	PZ161048
BRCL7	Broccoli	*Pseudomonas putida*	99.65	100	KX350025.1	PZ161049
BRCL8	Broccoli	*Stenotrophomonas* sp.	99.93	100	CP196972.1	PZ161050
BRCL9	Broccoli	*Pantoea agglomerans*	98.84	100	FJ357815.1	PZ161051
BRCL10	Broccoli	*Pseudomonas plecoglossicida*	100	100	NR_114226.1	PZ161052
BRCL11	Broccoli	*Pantoea vagans*	99.63	100	HG421010.1	PZ161053
BRCL13	Broccoli	*Pseudomonas putida*	100	100	PX401745.1	PZ161054
BRCL14	Broccoli	*Pseudomonas shirazensis*	99.93	100	OZ344942.1	PZ161055
BRCL20	Broccoli	*Pseudomonas shirazensis*	100	100	CP177047.1	PZ161056
BRCL21	Broccoli	*Enterobacter mori*	100	100	ON242152.1	PZ161057
BRCL22	Broccoli	*Pseudomonas shirazensis*	99.93	100	CP177040.1	PZ161058
BRCL24	Broccoli	*Pseudomonas oryzihabitans*	99.79	100	MZ350225.1	PZ161059
CLFR1	Cauliflower	*Pseudomonas versuta*	99.86	100	CP012676.1	PZ161060
CLFR2	Cauliflower	*Pseudomonas azotoformans*	100	100	PV370709.1	PZ161061
CLFR5	Cauliflower	*Pseudomonas putida*	100	100	KF843717.1	PZ161062
CLFR6	Cauliflower	*Pantoea agglomerans*	99.82	100	CP143418.1	PZ161063
CLFR8	Cauliflower	*Pseudomonas putida*	99.65	100	KF843717.1	PZ161064
CLFR10	Cauliflower	*Shewanella baltica*	97.93	100	ON248060.1	PZ161065
CLFR11	Cauliflower	*Shewanella baltica*	99.86	100	CP000753.1	PZ161066
CLFR12	Cauliflower	*Shewanella baltica*	100	100	ON248060.1	PZ161067
CLFR13	Cauliflower	*Acinetobacter soli*	98.31	100	CP016896.1	PZ161068
CLFR14	Cauliflower	*Acinetobacter beijerinckii*	100	100	AB859734.1	PZ161069
CLFR15	Cauliflower	*Pseudomonas monteilii*	100	100	FJ377542.1	PZ161070
CLFR16	Cauliflower	*Pseudomonas argentinensis*	99.65	100	LC769474.1	PZ161071
CLFR18	Cauliflower	*Pseudomonas canavaninivorans*	99.79	100	OQ130631.1	PZ161072
CLFR19	Cauliflower	*Enterobacter hormaechei*	99.93	100	CP085757.1	PZ161073
CLFR20	Cauliflower	*Phytopseudomonas argentinensis*	99.93	100	NR_043115.1	PZ161074
CLFR23	Cauliflower	*Pseudomonas graminis*	98.65	100	OP986399.1	PZ161075
CLFR24	Cauliflower	*Pseudomonas fluorescens*	99.86	100	AY538264.1	PZ161076
CLFR25	Cauliflower	*Shewanella baltica*	99.93	100	ON248060.1	PZ161077
CLFR26	Cauliflower	*Pseudomonas putida*	99.93	100	LC571940.1	PZ161078
CLFR28	Cauliflower	*Acinetobacter beijerinckii*	99.72	100	PQ148841.1	PZ161079
CLFR29	Cauliflower	*Pseudomonas fulva*	100	100	CP064946.1	PZ161080
CLFR31	Cauliflower	*Acinetobacter* sp.	100	100	AF336348.1	PZ161081
CLFR32	Cauliflower	*Pseudomonas putida*	99.79	100	CP026115.2	PZ161082
CLFR33	Cauliflower	*Pseudomonas putida*	100	100	PX401745.1	PZ161083
CLFR34	Cauliflower	*Shewanella putrefaciens*	99.79	100	KC951912.1	PZ161084
CLFR35	Cauliflower	*Pseudomonas* sp.	99.86	100	PP230929.1	PZ161085
CLFR36	Cauliflower	*Bacillus siamensis*	100	100	LC873067.1	PZ161086

## Data Availability

The 16S rRNA gene sequences of the bacteria isolated from broccoli and cauliflower in this study have been deposited in NCBI GenBank under the accession numbers PZ161046–PZ161086. Other datasets generated and/or analyzed during the current study are available from the corresponding author upon reasonable request.
